# Physical health and the complex role of PTSD symptoms in obesity: evidence from an Italian cohort of maltreated children and adolescents

**DOI:** 10.3389/fpubh.2026.1749018

**Published:** 2026-03-05

**Authors:** Elisa Fucà, Giulia Lazzaro, Stefania Falvo, Sara Passarini, Veronica Sperandini, Valentina Maria Mongiovì, Micol Viel, Benedetta Gadola, Federica Giovanniello, Deny Menghini, Paola De Rose, Stefano Vicari

**Affiliations:** 1 Child and Adolescent Neuropsychiatry Unit, Bambino Gesù Children's Hospital, IRCCS, Rome, Italy; 2 Department of Dynamic and Clinical Psychology and Health Studies, Sapienza University of Rome, Rome, Italy; 3 Department of Life Science and Public Health, Catholic University of the Sacred Heart, Rome, Italy

**Keywords:** body mass index, neglect, overweight, physical abuse, sexual abuse, trauma

## Abstract

**Background:**

Childhood maltreatment represents a significant risk factor for both mental and physical health problems, yet the interplay betyween post-traumatic stress disorder (PTSD) symptoms and obesity in pediatric populations remains underexplored. This study examined the distribution of selected physical health problems in a large cohort of maltreated children. Moreover, it investigated the potential mediating role of psychopathological symptoms in the relationship between PTSD symptoms and body mass index (BMI).

**Methods:**

This was a retrospective, cross-sectional study including 307 children and adolescents (aged 3–18 years) with documented histories of maltreatment. Data were collected from a file review of children and adolescents referred for a clinical evaluation at the Neuropsychiatry Unit of a pediatric Hospital.

**Results:**

Overweight/obesity was the most frequent condition (35.2%), followed by respiratory infections (9.8%), gastrointestinal symptoms (6.8%), and headaches (5.5%). Age emerged as a significant predictor of BMI, with older children showing a higher likelihood of overweight or obesity. Mediation analysis confirmed a partial indirect effect of PTSD symptoms on BMI through internalizing problems, alongside a direct negative effect on BMI, indicating the coexistence of opposing pathways linking maltreatment to weight outcomes. However, after controlling for age, PTSD symptoms only remained significantly associated with internalizing problems.

**Conclusions:**

These findings highlight the heterogeneity of children's responses to adversity, with some exhibiting weight loss, while others develop internalizing symptomatology associated with weight gain. Therefore, the assessment of internalizing symptoms when evaluating obesity risk in maltreated children and adolescents may be crucial. Integrating mental and physical health interventions is essential for preventing long-term adverse outcomes in this vulnerable population.

## 1 Introduction

The expression “adverse childhood experiences” (ACEs) refers to a heterogeneous group of potentially traumatic events including direct (e.g., abuse and neglect) and indirect (i.e. parental events that impact a child—such as parental incarceration, mental illness, suicide, or divorce) types (1). A meta-analysis of 19 studies published between 2006 and 2018 reported that the prevalence rates of one or more ACEs were 58.4% in North America and 42.2% in Europe (2). In Italy, it has been estimated that 193 children per 1,000 under the care of social services are subjected to a form of maltreatment (3). As for the types of abuse, the survey reveals that nearly half of the abused children (40.7%) experience a form of abuse related to inadequate caregiving. Witnessing domestic violence (32.4%) is the second most common form of abuse recorded: one in three abused children is a witness to domestic violence within the family. Another study involving 312 young adults aged 18–24 years reported a high incidence of emotional abuse (62%) followed by physical abuse (44%) and sexual abuse (18%) (4).

The long-term consequences of ACEs on mental health have been extensively studied: the exposure to childhood maltreatment is linked to a twofold increase in the risk of developing diagnosed mental health conditions, and more than doubles the likelihood of requiring prescription medications for their treatment (5). Across the lifespan, the exposure to ACEs is associated with higher prevalence of mental disorders in developmental age that may persist into adulthood (6, 7). In particular, significant associations have been documented with depression, PTSD (8), anxiety, psychosis (9), conduct/behavioral problems, and substance abuse (9–11). Some authors also suggest that the exposure to ACEs could be a predictor for the onset and development of personality pathology (12), and in the worst cases, also for suicide (13). Among these outcomes, PTSD has been identified as one of the most clinically relevant trauma-related conditions following childhood maltreatment, often co-occurring with internalizing psychopathological symptoms such as anxiety and depression.

A growing body of evidence indicates that the impact of ACEs extends beyond psychological consequences, contributing significantly to adverse physical health outcomes as well. Indeed, ACEs are linked to negative outcomes for health and development across short-, medium-, and long-term timelines. Their impact can be compounded, particularly during key windows of vulnerability and heightened developmental plasticity (14). Studies investigating the physical health-related consequences of ACEs in adulthood indicated higher risk to develop obesity (15)—in particular for women (16), diabetes, migraine, asthma, respiratory disease, chronic pain, and a wide range of more generic somatic symptoms (11, 17). Regarding pediatric age, there is some evidence that children and youth with special health care needs are more likely to have experienced ACEs (18). Indeed, children exposed to additional psychosocial stressors are more frequently affected by a wide range of common childhood illnesses, such as cute upper respiratory infections, intestinal infectious diseases and asthma, with a pattern indicating that greater exposure is associated with increased health risks (19). A study involving a sample of 3,955 adolescents found that physical maltreatment was related to an increase in the frequency and intensity of headaches in adolescents with migraines (20). Another research involving 48,567 child participants of 6 to 17 years of age reported that children and adolescents exposed to ACEs had increased risk for chronic pain (21).

Among the numerous physical health consequences linked to ACEs in pediatric age, obesity has emerged as one of the most extensively examined in literature. A systematic review including 24 studies found evidence indicating a link between ACEs and an increased risk of obesity in children (22). The authors reported that female children may be more vulnerable to the obesity-related impacts of ACEs compared to males and that, among the various types of ACEs, sexual abuse appears to exert a particularly strong influence on childhood obesity. Additionally, the presence of multiple concurrent ACEs may further elevate the risk of developing obesity during childhood (22). Intriguingly, symptoms of PTSD have been reported to fully mediate the positive relationship between ACEs and symptoms of eating disorders (23). These findings point to the possibility that trauma-related psychological symptoms may play a central role in shaping weight-related outcomes following early adversity.

It is important to note, however, that the mental and physical health consequences of childhood maltreatment are deeply interconnected. Psychological distress can contribute to the development and progression of physical health problems, both in childhood and later in life, while physical symptoms may, in turn, exacerbate mental health difficulties. This bidirectional relationship underscores the need for an integrated perspective when examining the impact of ACEs, and suggests that PTSD symptoms and associated internalizing psychopathology may constitute important pathways linking early adversity to alterations in BMI during development.

Given the considerable impact on both mental and physical heath, it is not surprising that ACEs determine consequences not only at individual level, but also on societal costs. For instance, a recent study estimating the health and financial burden of ACEs in the UK found that lifetime costs for childhood exposure to maltreatment and/or parental domestic violence and abuse, were £71,309 per child (non-fatal exposure), and £1,292,377 per childhood maltreatment fatality, with £27.8 billion projected costs (24). The authors also found that the total costs for exposure to parental domestic violence and abuse alone was £1.0 billion (24). In light of these substantial individual and societal costs, there is a pressing need for further research aimed at assessing the prevalence and nature of physical health conditions among children exposed to ACEs, as well as the psychological mechanisms through which these conditions may emerge. Such data are essential not only to better understand the full scope of the public health burden, but also to inform early intervention strategies and guide resource allocation for prevention and care.

Despite growing evidence linking ACEs to both mental and physical health outcomes, there remains a limited understanding of how physical health problems—particularly obesity—manifest across different developmental stages in children exposed to ACEs. Most existing studies either focus on adult populations or fail to disaggregate findings by age, making it difficult to identify critical windows for intervention. Furthermore, few investigations have explored how obesity in this population interacts with PTSD symptoms and psychopathology, leaving a gap in our understanding of the mechanisms through which early adversity may influence physical health trajectories. In particular, little is known about whether and how internalizing psychopathological symptoms mediate the association between PTSD symptoms and overweight/obesity in pediatric populations.

For these reasons, the aim of the present study was twofold: first, to examine the distribution of selected physical health problems in a large cohort of maltreated children, with a specific focus on overweight/obesity; and second, to investigate the potential mediating role of psychopathological symptoms in the relationship between PTSD symptoms and BMI. To the best of our knowledge, this is the first study addressing these issues in an Italian cohort involving only individuals in pediatric age who experienced ACEs. By addressing these aims, the study seeks to shed light on whether specific patterns of psychological distress contribute to the development of obesity in children with a history of adversity, and to identify possible targets for early, integrated interventions that encompass both mental and physical health.

## 2 Materials and methods

### 2.1 Participants and procedures

The study cohort was composed of 307 psychopharmacological treatment-naïve children and adolescents aged 3–18 years [age: 9.67 ± 4.17 years; 55.7% females, 44.3% males; 24.5% preschoolers, 43.6% school-age children and 31.9% adolescents] with a documented history of maltreatment referring for a behavioral evaluation at the Child and Adolescent Neuropsychiatry Unit of a pediatric hospital between 2019 and 2024.

This was a retrospective, cross-sectional study. Data were collected from a file review of children and adolescents referred for a clinical evaluation at the Hospital. Due to the retrospective design, data were collected from the hospital records and clinic charts and the de-identified data were analyzed. Cases were identified via a database including computer-generated list of all eligible cases. Data were originally extracted manually from individual medical charts. Data extracted included demographics (i.e. age, sex), BMI, selected episodic and chronic illness (i.e., gastrointestinal symptoms including gastroesophageal reflux, constipation, diarrhea, and abdominal pain; headache; autoimmune disorders including celiac disease, Crohn's disease, lupus, thyroid disorders; respiratory infections including asthma, recurrent tonsillitis, chronic rhinitis) and psychological measures (see “measures” section). Exclusion criteria were as follows: age < 3 or >18 years; the presence of genetic syndromes; history of maltreatment was substantiated by social services and/or judicial authorities—cases in which the history of maltreatment was self-reported but not officially documented were excluded. All participants were referred directly by hospital emergency departments or local child protection services, having already been identified as exposed to trauma-related experiences requiring clinical attention. These referrals were specifically made for the purpose of conducting a comprehensive neuropsychological and psychopathological assessment at our institution. Notably, our hospital employs a standardized protocol for the systematic screening and documentation of ACEs, which is integrated into the medical records of all patients, regardless of the primary reason for referral. Within this dedicated section, clinicians are required to document the presence of physical abuse, sexual abuse, domestic violence, neglect, or other forms of maltreatment.

Medical comorbidities were assessed through a review of patients' medical records, conducted to ensure accurate identification and documentation of relevant clinical conditions. Overweight was defined as a BMI at or above the 85th percentile and below the 95th percentile, whereas obesity was defined as a BMI at or above the 95th percentile (25).

All caregivers signed a written informed consent for data use for research purposes and a privacy statement that ensures that data will be kept confidential. The study was conducted according to the guidelines of the Declaration of Helsinki and was approved by the Local Ethical Committee (practice no 3188/2023, prot. N.827, NPI 3–03-2023).

#### 2.1.1 Descriptive characteristics of participants

Consistent with the aim of the study, we selected 307 children and adolescents presenting a history of maltreatment. Among them, 33% had a primary diagnosis of Adjustment Disorder, 22% of PTSD, 12% of Anxiety Disorders, 4.3% of Oppositional Defiant Disorder, 4% of Conduct Disorder, 3.2 % of Depressive Disorders, 2.9% of Disruptive mood dysregulation disorder, 0.3% of Complex PTSD, 0.3% of Obsessive-Compulsive Disorder; the remaining 18% only exhibited subthreshold psychopathological symptoms. All diagnoses were based on the criteria of the Diagnostic and Statistical Manual of Mental Disorders, Fifth Edition, DSM-5 (26) for patients who underwent the clinical assessment from 2019 to 2021 and DSM-5-TR (27) for patients who underwent the clinical assessment from 2022 to 2024, with the exception of the diagnosis of Complex PTSD who based on the ICD-11 criteria. The diagnostic process was carried out by neuropsychiatrists and developmental psychologists throughout a clinical examination based on the observation of the patient, the developmental history, and the semi-structured interview—the Kiddie Schedule for Affective Disorders and Schizophrenia—Present and Lifetime version, K-SADS-PL DSM-5 (28).

Developmental history and information were collected via clinical examination by an experienced clinician. In the current study, we considered the following information: neuropsychiatric family history, substance use during pregnancy, physical violence during pregnancy, weeks of gestation (classified as “term birth” ≥37 weeks of gestation or “preterm birth” < 37 weeks of gestation, according to the WHO and the International Federation of Gynecology and Obstetrics), birth weight (classified as “low birth weight”, i.e. under 2,500 grams; “normal birth weight”, i.e. 2,500–4,000 grams; or “high birth weight”, i.e. over 4,000 grams), mode of delivery, type of ACE experienced, and the presence of ongoing psychopharmacological treatment at the time of admission for psychological evaluation. Information on neuropsychiatric family history, substance use during pregnancy, physical violence during pregnancy, and ongoing psychopharmacological treatment at the time of admission are summarized in Table 1.

**Table 1:** Neuropsychiatric family history, substance use, pregnancy violence, and treatment at admission.

**Anamnestic information**	**Yes**	**No**	**No information**
Family history of neuropsychiatric disorders	30.3%	37.8%	31.9%
Substance use during pregnancy	2.3%	72%	25.7%
Physical violence during pregnancy	1.6%	84%	14.4%
Ongoing psychopharmacological treatment	15.3%	84.7%	–

In addition to the information summarized in Table 1, we also considered several other factors. These include the weeks of gestation, birth weight, and the type of ACE experienced. Specifically, 68.7% of participants were classified as having had a term birth, 9.4% were born preterm, and for the remaining participants, information on gestational age was not available. In terms of birth weight, 8.5% of participants were categorized as having a low birth weight, 60.5% as having normal birth weight, and 4.2% as having high birth weight. In terms of mode of delivery, 47.2% of participants were delivered by spontaneous vaginal birth, the 34.5% were delivered by cesarean section, while for 18.3% no data were available.

Regarding the type of abuse, 8.8% of participants experienced physical abuse, 12.7% experienced sexual abuse, 64.2% experienced neglect and emotional abuse, and 14.3% experienced multiple ACEs. Of note, a total of 82.7% of participants experienced maltreatment reported having experienced maltreatment within the family, 9.4% had been victims of maltreatment or abuse perpetrated by non-family members or occurring outside the domestic setting, while the remaining 7.9% experienced both forms of ACE.

### 2.2 Measures

All the tests listed and described below were administered during routine clinical activities.

#### 2.2.1 Trauma symptoms

Trauma Symptom Checklist for Young Children (29). The TSCYC is a 90-item caregiver-report measure of children's trauma- and abuse-related symptomatology. It contains two validity scales and eight clinical scales [Post-traumatic Stress-Intrusion, Post-traumatic Stress-Avoidance, Post-traumatic Stress-Arousal, Post-traumatic Stress-Total, Sexual Concerns, Dissociation, Anxiety, Depression, and Anger/Aggression, as well as an item assessing hours per week of caretaker contact with the child. The questionnaire was administered to a subgroup of caregivers (*n* = 182).

#### 2.2.2 Psychopathological screening

The CBCL (30) is a widely used parent-report questionnaire that contains 113-item evaluating the child's behaviors and emotions during the preceding 6 months on a three-point Likert scale. In the present study, we considered the T-scores of the broadband scales (internalizing problems, which incorporates anxious/depressed, withdrawn/depressed, somatic complaints; externalizing problems, which incorporates rule-breaking behavior, and aggressive behavior; total problems). According to the cut-off thresholds of Achenbach and Rescorla (2001), for these scales T-scores >63 are classified as clinically relevant, T-scores between 60 and 63 are classified as borderline, and T-scores < 60 indicate non-clinical symptoms.

### 2.3 Statistics

All statistical analyses were performed using SPSS version 28 (IBM Corp., Armonk, NY, USA). Descriptive statistics were calculated for health conditions and their characterization (age, gender, mono- vs. poly-victimization and type of abuse). Categorical variables were summarized as frequencies and percentages.

Associations between BMI and age was further examined using logistic regression. Age was treated as a continuous predictor (X) in the regression model, while the presence of obesity/overweight were analyzed as categorical outcome (Y). Odds ratios (ORs) with 95% confidence intervals (CIs) were reported to estimate the strength of associations.

**Figure 1:**
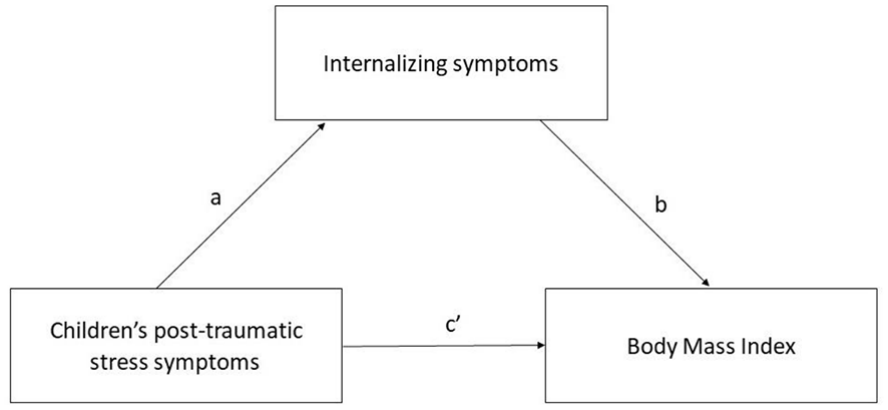
This figure illustrates the hypothesized mediation model, where children's post-traumatic stress symptoms represent the independent variable (X), BMI the dependent variable (Y), and children's internalizing symptoms the mediator (M).

The correlations between quantitative variables were analyzed using Pearson's r coefficient (*r*). A mediation model was conducted using PROCESS macro version 3.5 for SPSS (Hayes, 2018; Model 4), with TSCYC PTS TOT as the independent variable (X), BMI as the criterion (Y), and CBCL internalizing problems as the mediator (M). Indirect effects were estimated using 5,000 bootstrap samples with 95% bias-corrected confidence intervals. Figure 1 summarizes the hypothesized mediation model. Significance was determined if the 95% confidence interval did not include zero. Since age might be a significant predictor of BMI, the mediation model was further run with age as a covariate. Additional exploratory analyses testing sex as a moderator of the PTSD–BMI association were conducted and are reported in the Supplementary materials.

All tests were two-tailed, and a significance level of *p* < 0.05 was adopted for all analyses.

## 3 Results

### 3.1 Distribution of health co-occurrences

Among the total sample, obesity/overweight was the most prevalent health condition (*n* = 108, 35.2%), followed by respiratory infections (*n* = 30, 9.8%), gastrointestinal symptoms (*n* = 21, 6.8%), and headache (*n* = 17, 5.5%). In addition, autoimmune disorders (*n* = 7, 2.3%) were barely documented.

### 3.2 Characterization of obesity/overweight condition

Given that obesity and overweight were the most frequently reported conditions, we proceeded to characterize in depth their distributions across demographic features, mono- v*s*. poly-victimization and type of abuse.

*Demographic features*. First, age was a significant predictor of obesity/overweight (β = 0.091, OR = 1.095, 95 % CI [1.034, 1.160], *p* = 0.002), indicating that the likelihood of being overweight or obese increased significantly with age. Namely, each 10-year increase in age was associated with an approximately 9.5% higher likelihood of being overweight/obese compared to normal weight. To provide an overview of the distribution of overweight/obesity across age groups, we reported prevalence estimates in preschoolers (< 5 years), primary school children (6–11 years), and pre-adolescents/adolescents in secondary school (>12 years). Overweight/obesity was observed in 20.0% (*n* = 15 out of 75) of preschoolers, 35.1% (*n* = 47 out of 134) of primary school children, and 46.9% (*n* = 46 out of 98) of pre-adolescents/adolescents. With respect to gender, overweight/obesity condition (*n* = 108) encompassed 60.2% of females (*n* = 65), and 39.8% of males (*n* = 43).

*Mono—vs. poly-victimization and type of abuse*. With respect to the number of victimizations, overweight/obesity was observed in 34.6% (*n* = 91 out of 293) of participants with single abuse and 38.6% (*n* = 17 out of 44) of those with poly-abuse. Considering the type of abuse, the prevalence of overweight/obesity was 48.1% (*n* = 13 out of 27) among participants with physical abuse, 35.9% (*n* = 14 out of 39) with sexual abuse, 32.5% (*n* = 64 out of 197) with emotional abuse/neglect, 36.4% (*n* = 12 out of 33) with physical and emotional abuse, 33.3% (*n* = 1 out of 3) with physical and sexual abuse, and 50.0% (*n* = 4 out of 8) with sexual and emotional abuse.

### 3.3 Relation between PTSD symptoms, BMI, and psychopathology

First, Pearson's correlations between TSCYC PTS TOT and BMI revealed no significant direct association between PTSD symptoms and BMI (*r* = −0.098, *p* = 0.188). Second, Pearson's correlations between TSCYC PTS TOT and CBCL Broadband scales documented a significant association between PTSD symptoms and CBCL Internalizing Problems (*r* = 0.239, *p* = 0.001), indicating that PTSD symptoms increase, as internalizing problems increase. No correlation between TSCYC PTS TOT and CBCL Externalizing Problems (*r* = 0.057, *p* = 0.443) emerged. BMI was further associated with CBCL Internalizing Problems (*r* = 0.156, *p* = 0.007), and CBCL Externalizing Problems (*r* = 0.145, *p* = 0.011), meaning that BMI increase, as internalizing/externalizing problems increase. Overall, such correlations suggest a potential indirect pathway linking PTSD symptoms to BMI via internalizing problems.

In the first step, to formally test this potential indirect effect, a mediation model was conducted with TSCYC PTS TOT as the independent variable (X), BMI as the criterion (Y), and CBCL Internalizing Problems as the mediator (M). Results documented that the direct effects of TSCYC PTS TOT on BMI was negative and significant (*B* = −0.034, *p* = 0.036). Notably, while the previous analysis reported a non-significant direct correlation between PTSD symptoms and BMI, the mediation analysis revealed that the indirect effect of TSCYC PTS TOT on BMI through CBCL Internalizing Problems was positive and significant (*B* = 0.013, 95% bootstrap CI [0.002, 0.031]), indicating a partial mediation (for more details, see Table 2) which explains the 6.7% of the variance. Standardized coefficients (β) were reported as indicators of effect size.

**Table 2:** Direct effects between variables in the hypothesized mediation model.

**Pathways**	**Mediation model**				
	***B***	β	***SE***	***t***	***p***
a	0.14	0.22	0.04	3.32	0.001
b	0.09	0.21	0.03	3.33	0.001
c'	−0.03	−0.08	0.02	−2.12	0.036

a = relation between PTSD symptoms (TSCYC PTS TOT) and internalizing symptoms (CBCL Internalizing Problems); b = relation between internalizing symptoms (CBCL Internalizing Problems) and BMI; c' = relation between PTSD symptoms (TSCYC PTS TOT) and BMI. B = unstandardized regression coefficients; β = standardized regression coefficients.

In the second step, the mediation model was re-estimated including age as a covariate. After controlling for age, results documented that neither the direct effect of TSCYC PTS TOT on BMI (*B* = −0.01, *p* = 0.669) nor the indirect effect via CBCL Internalizing Problems (indirect effect: *B* = 0.006, 95% bootstrap CI [−0.002, 0.020]) remained statistically significant. TSCYC PTS TOT remained significantly associated with CBCL Internalizing Problems (*B* = 0.18, *p* < 0.001), and age was also a significant predictor of CBCL Internalizing Problems (*B* = 1.12, *p* < 0.001), with the model explaining 16.6% of the variance. Further, age emerged as the strongest predictor of BMI (*B* = 0.57, *p* < 0.001), whereas neither TSCYC PTS TOT nor CBCL Internalizing Problems were significantly associated with BMI.

## 4 Discussion

The first aim of the current study was to examine the distribution of selected physical health problems in a large cohort of maltreated children. We found that overweight/obesity (35.2%) and respiratory infections (9.77%) were the two most frequent medical co-occurrences.

These findings align with previous reports. The association between ACEs and respiratory conditions, indeed, has been previously reported in literature. Experiences of intense stressors, including community violence, are associated with an elevated likelihood of developing asthma (31). Moreover, the exposure to stress has been shown to precipitate asthma attacks in children within a short period (32). A study using data collected in 2007–2008 on 12,981 adults reported that retrospectively reported ACE was associated with a 9% increased risk of asthma and a 14% increased risk chronic bronchitis, and that such association is independent of respondent's mental health (33). A recent Mendelian randomization study, leveraging large-scale Genome-Wide Association Studies data from the UK Biobank, reported significant associations between childhood maltreatment and increased asthma risk, with consistent effects across both pediatric and adult phenotypes. By applying multiple statistical approaches and confirming the absence of pleiotropy, the authors strengthened the causal inference, emphasizing the relevance of early-life adversity in asthma etiology (34). ACEs may contribute to asthma and chronic bronchitis through multiple pathways. Stress-related alterations in brain development, stress-regulatory systems, and neuroendocrine–immune function can impair immune and cardiorespiratory processes, increasing vulnerability to respiratory infections and disease. Stress hormones and inflammation may further trigger airway constriction and mucus overproduction, raising asthma risk (35–37). Our findings underscore the relevance of respiratory conditions, alongside overweight and obesity, as common health problems among maltreated children. Together with prior evidence, these results suggest that ACEs may represent a potential risk factor for asthma and related respiratory disorders, possibly operating through both biological and stress-related pathways. This emphasizes the need for early prevention and monitoring strategies targeting respiratory health in vulnerable populations.

On the other hand, we found that overweight/obesity was the most frequent health co-occurrence in our sample. The WHO European Childhood Obesity Surveillance Initiative reported that 25% of children aged 7–9 years were living with overweight (including obesity) in 2022–2024(38). In Italy, 2023 data indicate that 19% of children aged 8–9 years are overweight, while 9.8% are living with obesity (39). These comparisons suggest that the prevalence of overweight and obesity among maltreated children in our cohort is higher than that observed in the general pediatric population, underscoring the potential impact of early adversity on physical health trajectories. However, such interpretations should be made with caution, as differences in study design, age range, and assessment methods may partly account for the observed discrepancies.

The link between ACEs and elevated risk of overweight and obesity likely arises from several putative mechanisms underlying this association. The first one refers to alteration to cortisol levels following exposure to ACE. Prolonged exposure to elevated cortisol levels may contribute to obesity through multiple biological pathways. It can stimulate appetite, particularly increasing the preference for calorie-dense foods; it may promote the formation and enlargement of fat cells, especially within visceral adipose tissue; and it can reduce thermogenesis in brown fat, thereby lowering overall energy expenditure (40). Other biological alterations explaining the link between ACEs and overweight/obesity may be identified in reward-related neural responsiveness (41), and mechanisms of cognitive or behavioral regulation (42)—as well as differences in weight-control strategies (43). An intriguing insight about the potential mechanisms linking ACEs to obesity derives from a study by Manassero and colleagues (44), which explored cognitive-emotional processes in obesity. Their findings indicate that individuals with obesity exhibit enhanced fear generalization, showing stronger autonomic responses to novel stimuli resembling previously learned threats. Although this study was conducted in adults, it suggests that altered threat evaluation and emotional dysregulation may represent additional pathways through which early-life adversity contributes to obesity. This perspective complements the biological mechanisms discussed above, highlighting that both physiological and cognitive-emotional processes may jointly influence the risk of overweight and obesity following childhood maltreatment.

In our sample, we did not reply previous results indicating that polyvictimization is associated with higher risk of obesity (10), since overweight/obesity was observed in 34.6% of participants with single abuse and 38.6% of those with poly-abuse. Methodological and sample-related factors may have influenced this result, and further research is needed to clarify these associations. However, one possible explanation is that even a single form of maltreatment, such as physical abuse, may be sufficient to confer an elevated risk of obesity in childhood.

Of note, age emerged as a statistically significant predictor of BMI, with older participants showing an increased likelihood of overweight and obesity. This aligns with literature reporting that the effect of ACEs on development of childhood obesity may take 2–5 years to manifest (22). Of note, the overall predictive performance of the model was limited, suggesting that additional variables and more complex modeling approaches may be needed to capture the multifactorial nature of weight-related outcomes. In this sense, psychopathology is a leading candidate factor explaining, at least in part, such association between age and BMI. Previous research has consistently shown that mental health problems such as depression, anxiety, and behavioral dysregulation are associated with unhealthy lifestyle behaviors, including emotional eating, sedentary patterns, and irregular sleep (45, 46), all of which contribute to weight gain over time; intriguingly, the relationship between mental health issues and unhealthy eating habits is bidirectional, with evidence suggesting that consumption of junk foods was associated with an increased hazard of developing depression in adults (47). Moreover, psychopathological symptoms may alter neuroendocrine functioning and stress-response system (48), thereby increasing vulnerability to metabolic dysregulation. Within maltreated populations, these mechanisms are likely to be amplified, as early adversity is a well-documented risk factor for both psychopathology and altered physiological stress responses. Thus, it is plausible that the age-related increase in overweight/obesity observed in our sample reflects the cumulative impact of prolonged exposure to psychopathology on health behaviors and biological systems.

This interpretation is corroborated by the results of the first mediation model tested. Indeed, we found that PTSD symptoms exert two opposing influences on BMI. On the one hand, higher levels of PTSD symptoms were directly associated with lower BMI. On the other hand, PTSD symptoms indirectly predicted higher BMI through their positive association with internalizing symptoms, which in turn were linked to increased BMI. Our results suggest the existence of different pathways linking ACEs to BMI in children and adolescents: a “direct pathway” through which PTSD symptoms are linked to lower BMI and an “indirect pathway” through which PTSD symptoms are linked to higher BMI via internalizing symptoms. This confirms and extends previous findings on school-age children and adolescence reporting that ACEs exhibit significant association with specific forms of disordered eating behaviors, namely skipping meals or fasting, low interest in food, and binge eating (49).

The coexistence of opposite pathways may reflect heterogeneity in clinical presentations: some children respond to ACEs with hyperarousal and appetite suppression (leading to lower BMI), while others develop internalizing symptoms that promote weight gain. Moreover, these distinct pathways may highlight individual differences in coping strategies (e.g., avoidance vs. comfort-seeking through food), which could explain why the same ACE exposure leads to divergent BMI outcomes. Our findings are consistent with the results of a longitudinal study involving 38,352 participants, which showed that PTSD is independently associated with a higher risk of weight gain and loss (50).

The direct association between PTSD symptoms and lower BMI may be accounted by several factors. First, alterations in the neuroendocrine (e.g., HPA axis hyperactivation, altered cortisol regulation) and autonomic nervous systems can have a direct impact on sleep, metabolic processes, and appetite, potentially leading to weight changes (51). In addition, food intake restriction may be also a coping strategy to avoid hyperarousal symptoms and distressing memories (52, 53); whether comparable strategies are often adopted by children and adolescents requires further investigation. Finally, somatic symptoms associated with PTSD, especially gastrointestinal disturbances (54, 55), may influence weight loss, although direct evidence for these mechanisms in pediatric samples is still scarce. Overall, these interpretations should be considered speculative and highlight the need for future developmentally informed research.

The “indirect pathway” through which PTSD symptoms are linked to higher BMI via internalizing symptoms can be supported by several mechanisms. On one hand, the link between ACEs and internalizing problems is well documented, with literature reporting that nearly 50% of people with depression report having experienced childhood maltreatment (56). Such link is rooted in genetic and epigenetic (57–59), biological (60), and psychological factors, such as self-compassion (61), and self-criticism (62). The role of other elements mediating this association, such as the microbe-gut-brain axis (63), deserves more research. It is worth to note that while PTSD symptoms were robustly associated with internalizing problems, their direct and indirect associations with BMI were no longer significant after adjusting for age, indicating that developmental factors account for the observed relationship with BMI. However, despite the crucial role of age, it is necessary to identify potential cognitive-behavioral coping, which may underly the association between internalizing problems and BMI. One of the leading candidates is emotional eating. Indeed, greater depression symptoms are associated with greater emotional eating in children and adolescents cross-sectionally and longitudinally (64); moreover, emotional eating in youth has been associated with anxiety symptoms (65) and social anxiety (66). It should be also mentioned that internalizing problems, which may lead to lethargy, fatigue, or social withdrawal, often affect exercise habits, reducing energy expenditure and contributing to weight increase (67).

Our findings in pediatric age provide useful insights to the current evidence from adults indicating that females are at a higher risk of obesity after ACEs than males (16). Similar to findings in adults, in our sample females were more represented than males in the overweight/obesity group. Intriguingly, these data seem to contrast with reports from general population, indicating higher prevalence of overweight among boys compared to girls (38). However, it is important to recognize that the present mediation model provides a partial account of the pathways influencing BMI, which are likely to be multifaceted and extend beyond the scope of the current analysis. Accordingly, additional exploratory analyses testing sex as a moderator and controlling for age (see Supplementary Materials) showed that sex did not significantly modify the associations between PTSD symptoms, internalizing problems, and BMI.

The results of the current study should be interpreted in light of several limitations. First, the wide age range of the participants may have introduced variability related to different developmental stages, which could have influenced the manifestation of health outcomes. Second, the lack of a control group makes it difficult to disentangle the specific contribution of ACEs from other potential risk or protective factors, and therefore limits the generalizability of our findings. Another limitation of this study is its retrospective chart review design, which may expose the findings to biases related to the completeness and quality of the available clinical documentation. Moreover, although all participants were psychopharmacological treatment-naïve, we cannot entirely exclude the concomitant use of non-psychotropic medications prescribed for other medical conditions (e.g., antihistamines for respiratory problems), which may have influenced weight outcomes. Finally, the cross-sectional nature of the design restricts the possibility of establishing temporal or causal relationships, preventing us from fully clarifying the directionality between ACEs, psychopathology, and physical health. Future longitudinal research with more homogeneous samples and appropriate control groups will be essential to confirm and extend these preliminary observations.

Despite these limitations, our findings offer valuable evidence on the association between ACEs and physical health in pediatric age, highlighting the specific role of psychopathology in shaping this relationship.

From a clinical perspective, these findings suggest that the exposure to ACEs do not exert a uniform, linear effect on body weight in children and adolescents. Some individuals may respond with weight loss or lower BMI, reflecting the direct impact of PTSD symptoms such as hyperarousal, avoidance, or appetite suppression. Others, however, may show an increase in weight, particularly when internalizing symptoms such as depression and anxiety become prominent, highlighting the mediating role of psychopathology. This underscores the importance of internalizing symptoms as a key factor: not all children exposed to ACEs will present with BMI alterations, but those with significant internalizing difficulties may be more likely to develop overweight or obesity. For clinical practice, this means that monitoring PTSD symptoms alone is insufficient; clinicians should also systematically assess the presence and severity of internalizing problems. In this context, BMI trajectories may serve as an indirect indicator of a child's response profile to ACEs, with weight loss being more characteristic of hyperarousal-driven presentations and weight gain more likely among those with internalizing psychopathology.

In conclusion, the current study provides further support to the notion that childhood maltreatment is a global public health threat. To overlook such evidence would mean condemning entire generations to trajectories of fragility and suffering. Thus, the evaluation and implementation of early interventions is not merely desirable—it is an ethical and social imperative, without which the developmental pathways of maltreated children risk becoming irreversible courses of pain.

## Data Availability

The raw data supporting the conclusions of this article will be made available by the authors, without undue reservation.
