# Supplementary material for: Physical health and the complex role of PTSD symptoms in obesity: evidence from an Italian cohort of maltreated children and adolescents
Source: Front Public Health. 2026 Mar 5;14:1749018. doi: 10.3389/fpubh.2026.1749018 (PMC12999874; doi:10.3389/fpubh.2026.1749018)
Supplement: Supplementary file 1 [file Supplementary_file_1.docx]

Supplementary Material

# Supplementary Analysis

To exploratory examine the moderator role of sex in the second mediation model, additional analyses were conducted. Specifically, a moderated mediation model (PROCESS Model 5) was estimated to test whether sex moderated the direct association between TSCYC PTS TOT and BMI, while CBCL Internalizing Problems were included as a mediator. Age was included as a covariate, whereas sex was included as a moderator. All analyses were conducted using 5,000 bootstrap samples to estimate confidence intervals.

# Supplementary Results

Results from the moderated mediation model indicated that TSCYC PTS TOT were significantly associated with CBCL Internalizing Problems after controlling for age (B = 0.18, *p* < 0.001), and age was also a significant predictor of CBCL Internalizing Problems (B = 1.12, *p* < .001). In the model predicting BMI, age emerged as the only significant predictor (B = 0.58, *p* < .001), whereas TSCYC PTS TOT (B = 0.07, *p* = .172), internalizing problems (B = 0.03, *p* = 0.345), and sex (B = 2.79, *p* = 0.159) were not significantly associated with BMI. Importantly, the interaction between TSCYC PTS TOT and sex was not statistically significant (*p* = 0.123), indicating no moderation effect. The overall model explained 25.5% of the variance in BMI. Consistently, conditional direct effects of TSCYC PTS TOT on BMI were not significant in either sex group, and the indirect effect of TSCYC PTS TOT on BMI via CBCL Internalizing Problems was not statistically significant (indirect effect: Β = 0.005, 95% bootstrap CI [−0.004, 0.018]).
